# Performance and usability evaluation of the INSTI HIV self-test in Kenya for qualitative detection of antibodies to HIV

**DOI:** 10.1371/journal.pone.0202491

**Published:** 2018-09-13

**Authors:** Priska Bwana, Lydia Ochieng’, Matilu Mwau

**Affiliations:** Kenya Medical Research Institute, Busia, Kenya; Waseda University, JAPAN

## Abstract

**Background:**

HIV testing is often undermined by lack of confidentiality, stigma, shortage of counselors and long distances to testing centers. Self-testing has the potential to circumvent these constraints.

**Objective:**

To determine the performance and usability characteristics of the INSTI® HIV-1/HIV-2 Self-Test.

**Methods:**

The performance evaluation was a cross sectional study and the usability a mixed methods study. For method comparison, Bioelisa HIV-1+2 Ag/Ab test was used as the reference test. When the test results were discrepant, results from Alere Determine™ HIV-1/2 and First Response HIV-1-2 Antibody tests were used for confirmation of status.

**Results:**

Sensitivity of the INSTI HIV Self-Test was 98.99% (95% CI 96.05–99.75%), and specificity 98.15% (95% CI 95.63–99.23%). The concordance was therefore 97.27%. A total of 354 participants took part in the usability study. Of those, 343 (98.00%) found instructions for use easy to follow, 330 (94.29%) found the finger prick device easy to use, 303 (86.57%) were confident while performing the test, 342 (97.71%) felt result interpretation was easy, while 304 (86.86%) declared results within the recommended five minutes. Three hundred and forty two (342, 97.71%) were willing to use the test again while 344 (98.29%) would recommend the kit to a sexual partner. None of the 350 participants quit the process at any stage. Three hundred and eighteen (318, 91.12%) participants felt the test needed no further improvement. All 91 lay users correctly identified cartridges that showed positive, negative and invalid results. Only 31 (34.07%) participants correctly identified weak positive dummy test results.

**Conclusion:**

The excellent performance and usability characteristics of INSTI HIV-1/HIV-2 self-test make the kit a viable option for HIV self-testing. To improve the identification of weak positive results, the manufacturer should indicate on the IFU that even a faint test spot should be interpreted as positive.

## Introduction

Kenya has an adult HIV prevalence rate of 5.4%, with approximately 1.6 million people living with HIV. Of these, approximately 53% are unaware of their HIV status. HIV testing and counseling (HTC) has been a major feature of Kenya’s HIV/AIDs response and this has seen the number of adults aged 15–64 years who tested for HIV increased from 37% in 2007 to 70% in 2014 and to 80% in 2015[1, 2]. This increase in first time HIV testing is significant, but as UNAIDS targets 90% of the people living with HIV knowing their status by 2020, national programs need to adopt innovative testing approaches that can reach the untested populations.

HIV testing landscape in Kenya has been rapidly changing. Previously, HIV testing was laboratory based and testing was done using Enzyme Linked Immunosorbent Assay (ELISA). In 2001, HIV testing and counseling was launched and client initiated approach was used [3]. This was followed by the adoption of a number of innovative approaches to HIV testing such as voluntary counseling and testing centers, targeted community-based HIV testing and door-to-door testing campaigns[4].Hospitals and health facilities also began incorporating provider initiated HIV testing and counseling (PITC) as part of routine health care to all patients[5].These approaches have seen the country increase the number of people testing for HIV annually from approximately 860,000 people in 2008 to approximately 9.9 million in 2015[6].

Numerous assays for rapid HIV antibody detection have been developed and are used for HIV screening and diagnosis in Voluntary Counseling and Testing centers (VCT), provider-initiated counselling and testing centers (PICT) and community outreach testing programs. These assays can be based on solid phase (lateral flow) immunochromatography[7], rapid (flow-through) immunofiltration or agglutination[8]The use of up to three different HIV rapid assays in adults in parallel algorithms has helped in ensuring wide-scale diagnosis and access to care[9]. In Kenya, the current national HIV testing algorithm is constituted of two different HIV rapid assays; a screening test and a confirmatory test. This guideline does not recommend use of a tie-breaker. Therefore, in case of inconclusive results, samples are referred to the laboratory for further analysis.[10].

Routine use of HIV rapid kits has promoted and encouraged testing [11] and as a results, has increased uptake of HIV testing in various settings., However, key populations such as adolescents, men who have sex with men, and commercial sex workers form a disproportionate number of those who do not know their status. Also, there is a significant disparity between the number of men and women who test for HIV. Additionally, constraints such as lack of privacy, long waiting times in facilities, social stigma and the inconvenience and personal costs involved in accessing testing services have been associated with the current HIV testing approaches[12].

HIV self-testing (HIVST) is a new strategy, with the potential to address and overcome these challenges [12]. It is a process in which an individual collects his /her own sample (blood or oral fluid), performs a HIV rapid diagnostic test and interprets the result in private[13].

In order to reach the unreached HIV infected people and link them to care and treatment, the National AIDs and STI control program (NASCOP) in Kenya prepared and launched the HIV testing service guideline in 2015[10].This guideline recognized the potential of HIV self-testing to act as a catalyst in increasing access and coverage of HIV testing. In 2017, the same program launched HIV self-testing operational guidelines, following the roll out of HIVST[14].

In Kenya, the OraQuick self-test (OraSure Technologies) which uses oral fluid, and the Atomo HIV Self-test (Atomo Diagnostics Pty Ltd., Sydney, Australia) a blood based test, have been approved for use [14–16], Currently, OraQuick self-test are readily available in the market. The pool of HIV self-tests kits approved and available for use in Kenya is small. Therefore, the national HIV program is continuously looking for HIV tests to help improve access to HIV care and testing. INSTI® HIV Self-Test has the potential to meet this need.

INSTI HIV-1/HIV-2 Antibody Test (BioLytical® Laboratories Inc., Richmond, B.C., Canada) is a rapid in vitro qualitative test for the detection of antibodies to HIV 1/2 in human whole blood, serum and plasma samples within 5 minutes. It is based on flow through technology and the membrane contains a test spot blotted with recombinant peptide analogues of the HIV-1 gp41 and HIV-2 gp36 epitopes[17].Its concordance has been reported as 99%[18–21]. It has a sensitivity of 99.8–100% and a specificity of 99.5–99.8%[22, 23]. When the test was compared to FDA-approved antibody-based lateral flow rapid tests, it showed a sensitivity of 99.84% and specificity of 99.80%, and when used for detection of early sero-conversion HIV Infection it showed a sensitivity 69.4% (95% CI 54.6–81.8%)[24–26]. The INSTI® HIV Self-Test received its CE marking from European Union regulators in July 2016 [20, 27]. In December of the same year, BioLytical Laboratories Inc. announced the expansion of INSTI HIV Self-test to Africa with a lower cost version, following the release of new guidelines on HIV self-testing by WHO [19]. Currently, the self-test is commercially available in the UK, France and other European countries [20, 21, 27].

Kenya is continually evaluating new technologies for HIV testing in order to maintain a robust pipeline in support of the national program. This study sought to determine the sensitivity, specificity and usability of INSTI HIV Self-Test (BioLytical Laboratories Inc., Richmond, B.C., Canada) for qualitative detection of antibodies for HIV using capillary blood in field settings and whole blood in laboratory evaluations in Kenya.

## Materials and methods

### Study design

The performance evaluation of the INSTI HIV Self-test was cross sectional study of consenting adults in Western Kenya using convenience sampling. Villages were selected based on how far they were from the health centre/health facility. Village chiefs and County administration informed and mobilized their respective villages about voluntary HIV testing, within which a study of the INSTI HIV self-test was nested. The testing exercise was conducted at market centers and village squares. For the second part of the performance study, known HIV positive patients were recruited from clinics in the study area.

For method comparison, bioelisa HIV-1+2 Ag/Ab (Biokit SA, Barcelona, Spain) was used as the reference test. The null hypothesis was that the INSTI® HIV Self-Test is as sensitive and as specific as bioelisa HIV-1+2 Ag/Ab test for HIV. The minimum sample size for the study was determined to be approximately 200 HIV-positive and 200 HIV-negative specimens to provide 95% confidence intervals of less than ± 2% for an estimated sensitivity of 98% and a specificity of 98% and a HIV prevalence rate of 5.9% [28].

The usability study was a mixed methods study, was not designed for hypothesis testing, and was driven by the quantitative methods comparison study. Those who consented were observed as they conducted the test and the findings recorded. They were also requested to fill in a short questionnaire. The questionnaire was evaluating perception of the test. For readability, the first 100 participants from the methods comparison study who consented were given contrived (dummy) tests and asked to report what the various results were.

This study was reviewed and approved by the Kenya Medical Research Institute’s Scientific Ethical Review Unit (SERU) (KEMRI/SERU Protocol No. 2657). Since this was a research study, written informed consent was requested for both HIV testing and for study participation from every participant. A simple questionnaire was administered. The study was conducted according to the principles expressed in the Helsinki Declaration.

### Study participants

**Fig 1:**
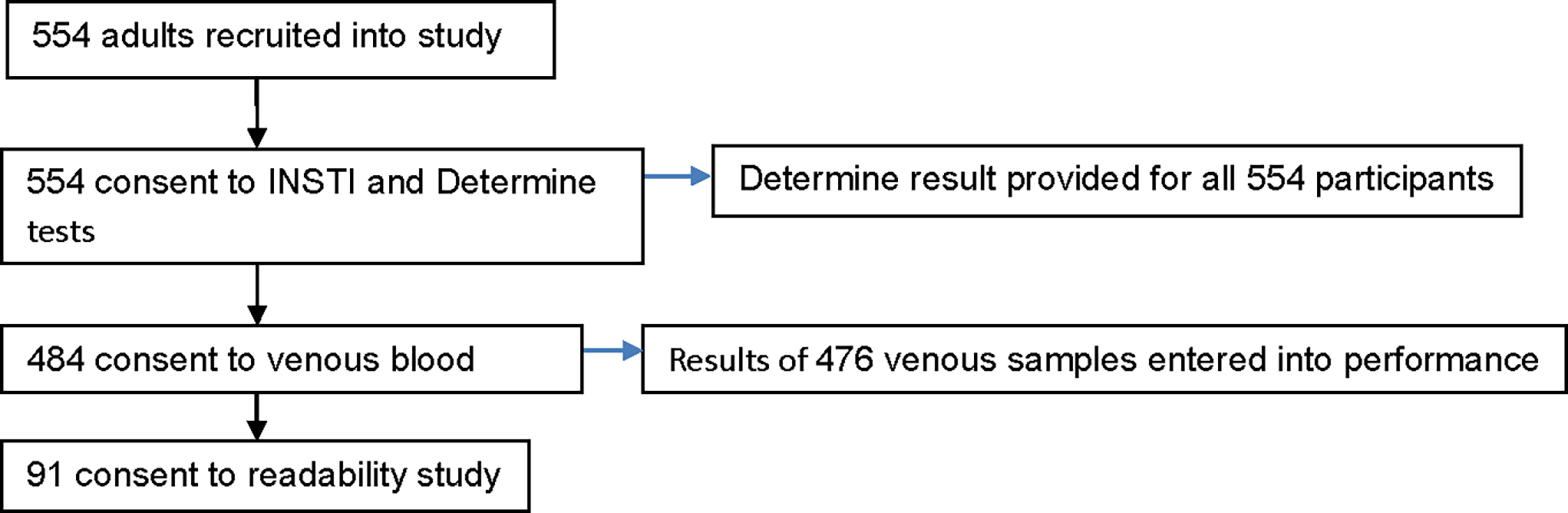
Flow diagram of participants in INSTI HIV self-test study.

The performance, acceptability, perception and ease of use of the INSTI HIV Self-Test was evaluated amongst 554 consenting adults at Matayos, Bumutiru, Khunyangu, Aterait and Asinge villages in Busia County, Western Kenya using a structured questionnaire administered by the observer. Of the 554, 484 participants consented to the venous blood draw while 91 consented to the readability study additionally (Fig 1).

### Field procedures

The kit manufacturer ensured that necessary training was provided to the field study team. Participants were supervised in person, with one participant per observer per testing period. Testing was conducted in private and each participant received an individual demonstration on how to perform the test and interpret the results as per the instructions for use (IFU) sheet, which was in the pouch. Although alcohol swabs are optional in self-testing using the INSTI HIV Self-test and were not provided by the manufacturer, it was determined by the study investigators from observation of the extent of soiling of hands by participants that the use of alcohol swabs was warranted.

**Fig 2:**
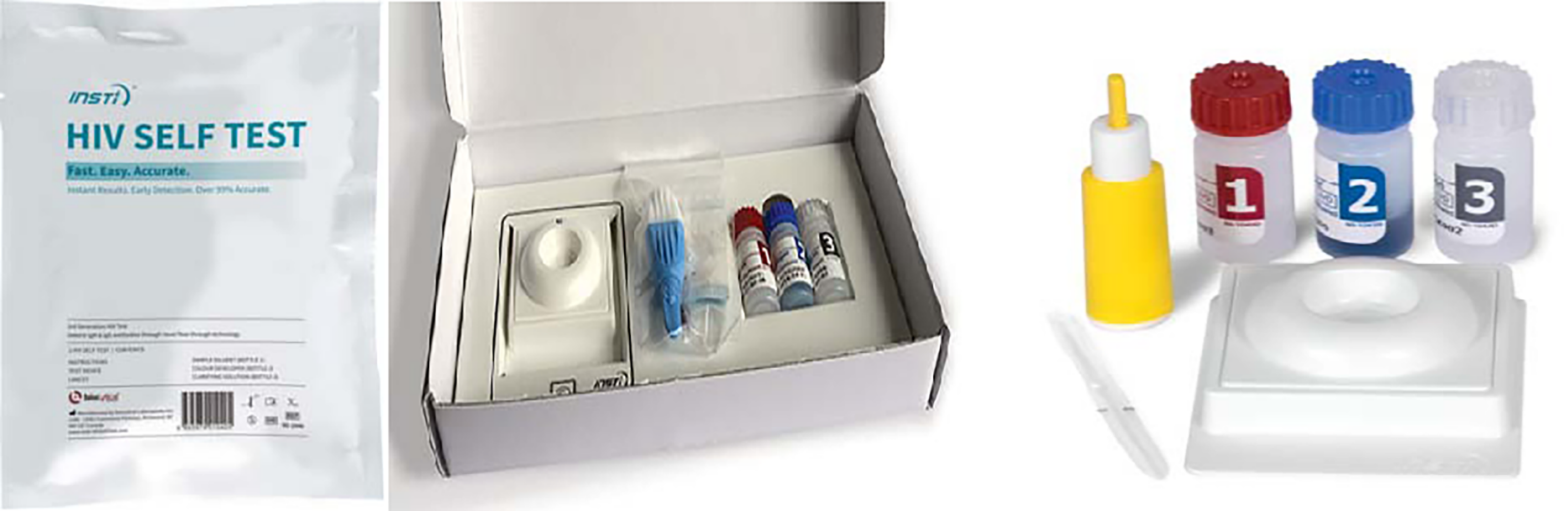
INSTI HIV-1/HIV-2 self-test (source www.biolytical.com).

Every participant was provided with the testing package, observed and evaluated by the observer using a questionnaire shown in annex 2 for completeness of each step as described in the IFU. All participants interpreted their own test results as per the results interpretation guide on the IFU. The sealed testing package, the test cartridge, the lancet and the diluent fluids are shown in Fig 2 below.

From the same finger-stick, the VCT counselor expressed an additional drop of blood and applied this on the Alere Determine™ HIV-1/2 Antibody (Alere, Waltham, MA, USA) test kit that was part of the routine testing algorithm. The participant was provided with this result and was counselled appropriately by the VCT Counselor who formed part of the team. A further venous blood sample was drawn and the blood sent to the laboratory for ELISA testing. Both participant and VCT counselor interpreted the HIVST result. The role of the counsellor was to identify any discordance: was no discordance in the results of this study. However, only the lay results were used for performance evaluation.

**Fig 3:**
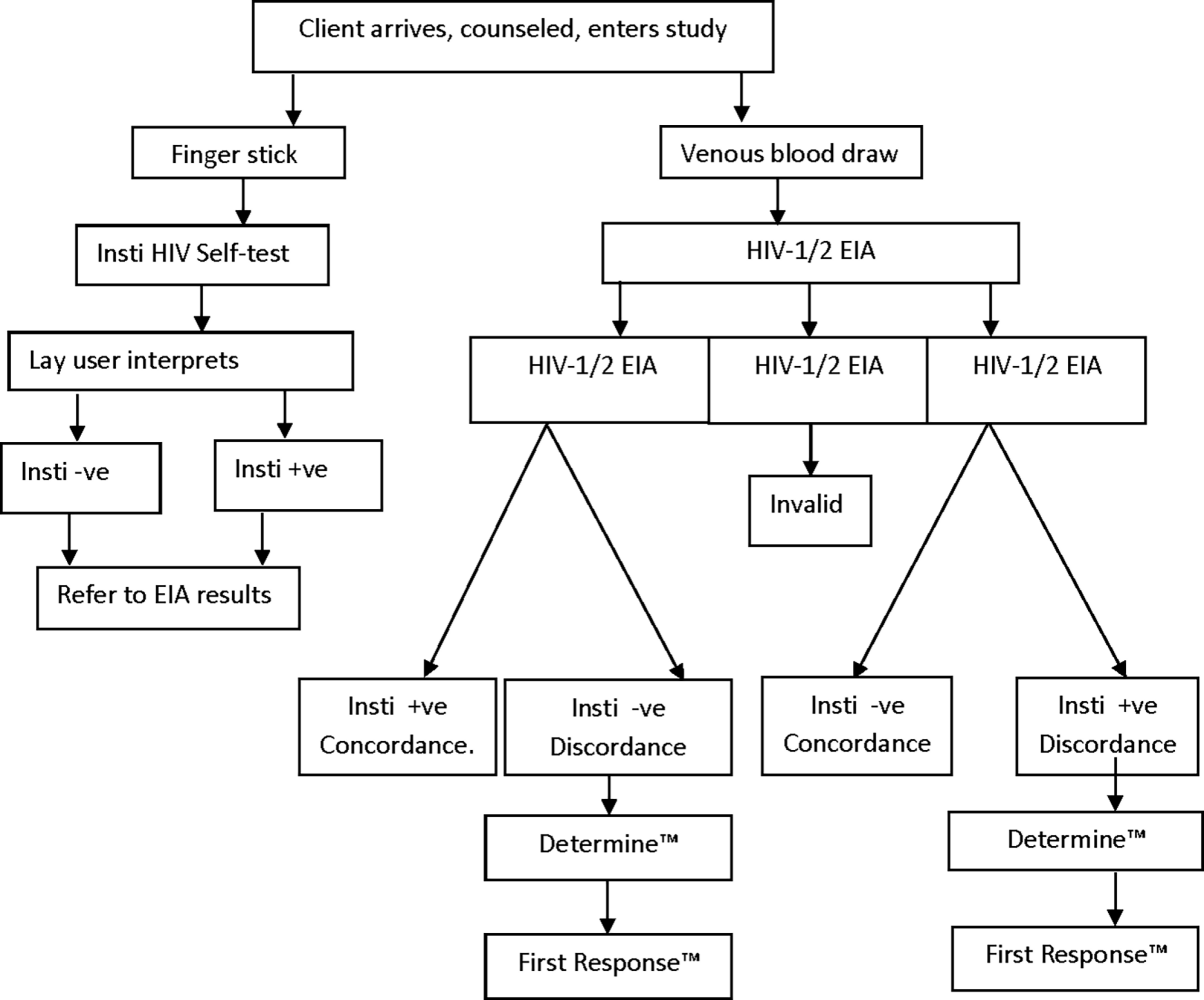
Workflow and outcomes interpretation in the facilities and the testing laboratory.

For perception and acceptance evaluation, a questionnaire was administered to the study participants before and after testing. The response from the participants was scored for evaluation. The complete workflow in the facility and the laboratory, including outcome interpretation, is shown in Fig 3.

### Laboratory procedures

#### Enzyme linked immunosorbent assays

The bioelisa HIV-1+2 Ag/Ab (Biokit SA, Barcelona, Spain) test was used as the reference for this study. It is a fourth generation ELISA test for qualitative detection of antibodies to HIV-1, HIV-2 and HIV-1 p24 antigen in human serum or plasma, considered as good as or even better than Enzygnost Anti-HIV 1/2 Plus (Siemens Healthcare Diagnostics) and Vironostika HIV Ag/Ab (bioMérieux) EIA, the current WHO prequalified benchmark assays[29]. The assay was run according to the manufacturer’s instructions. When the results of the INSTI HIV self-test and the reference test were discrepant, the results from the Alere Determine™ HIV-1/2 Antibody (Alere, Waltham, MA, USA) and First Response HIV-1-2 kit (Premier Medical Corporation Ltd., Kachigam, India) test were used for confirmation of true status.

### Ethical considerations

The evaluation was carried out in line with existing ethical guidelines and was approved by the Institutional Review Board of the Kenya Medical Research Institute (KEMRI/SERU Protocol No. 2657).

### Data analysis

Data was transferred from the data sheets into an excel database. The analysis was done using Stata/MP Version 14 for Mac OSX. Qualitative data on usability was coded, aggregated, analyzed for recurring themes and tabulated as percentages. Performance data was coded and aggregated; the relationship between INSTI HIV Self-test results and the HIV status of the study participants was evaluated in a two-way table and tested using the chi square test. Readability data was coded and described as percentage readability for each variable. Diagnostic accuracy was defined using sensitivity, specificity, likelihood ratio positive and concordance as previously described[30].

## Results

### Socio demographic characteristics of participants

A total of 354 study participants enrolled for this arm of the study; only complete data was used for each usability parameter. One hundred and forty-two (142, 40.57%) were female while 206 (58.86%) were male. Two (2, (0.57%) provided no data on their sex. Twenty-two (22, (6.29%) had university level education, 123 (34.14%) had secondary and 152 (43.43%) primary education. There was no data on education from 5 (1.43%) while 48 (13.71%) had received no education at all.

### Usability and perception of the INSTI HIV self-test

The median time to results was five (5) minutes. Three hundred and forty-three (343, 98.00%) participants used the instruction sheet, 2 (0.57%) did not while 5 (1.43%) did not answer the question. On ease of understanding, instructions were easy to comprehend to 339 (96.86%), while 7 (2.00%) recorded that it was not easy. Four (4, (1.14%) did not answer the question. Three hundred and forty-two (342, (97.71%) found interpretation of results to be easy, but 2 (0.57%) thought not. Six (6, (1.71%) provided no opinion.

Perception of the participants on willingness to recommend the kit to a sexual partner for use was also evaluated. Three hundred and forty-four, (344 (98.29%) were affirmative, 4 (1.14%) were negative, while 2 (0.57%) provided no data. These and other usability characteristics are summarized in Table 1 below.

**Table 1:** Usability characteristics and perception of the INSTI HIV self-test.

Characteristics	Female	Male	Total	Percent
**Total**	** 142**	** 206**	**348**	**100**
**Use of Instruction sheet**				
Yes	140	202	342	98
No	1	1	2	0.57
No data	1	3	4	1.43
**Ease of Understanding Instructions**				
Easy	139	199	338	96.86
Not easy	3	4	7	2
No data	0	3	3	1.14
**Ease of use of the test device**				
Easy	136	193	329	94.29
Not easy	5	10	15	4.29
No data	1	3	4	1.43
**Confidence in performing the self-test**				
Yes	121	180	301	86.57
No data	2	7	9	2.86
No	19	19	38	10.57
**Time to results**				
Five minutes or less	121	183	304	86.86
Six to ten minutes	12	9	21	2
Eleven to thirty minutes	2	4	6	6
No data	7	10	17	5.14
**Ease of results interpretation**				
Yes	140	201	341	97.71
No data	1	4	5	1.71
No	1	1	2	0.57
**Perception of waste generated**				
Little	112	169	281	80.57
Moderate	8	9	17	4.86
Too much	10	13	23	6.57
No idea	1	0	1	0.29
No data	11	15	26	9.04
**Suggestions for improvement**				
None	130	187	317	91.12
No data	4	9	13	4.29
Reduce bottles or liquids	1	0	1	0.57
Reduce the processes	3	3	7	2
Provide spectacles	0	1	1	0.29
Provide Braille	0	1	1	0.29
Avail the product	0	1	1	0.29
Sensitize public on product	0	1	1	0.29
Provide trainers	1	1	2	0.57
Provide counselors	1	1	2	0.57
**Willingness to use the test kit again**				
Yes	139	202	341	97.71
No	1	2	3	0.86
No data	1	2	3	1.14
Not sure	1	0	1	0.29
**Willingness to recommend the kit to a sexual partner for use**				
Yes	139	204	343	98.29
No data	0	1	1	0.57
No	3	1	4	1.14

Other additional desirable usability characteristics for the INSTI HIV 1/2 Self-Test were also evaluated. They included: ease of opening of the pouch, uncapping the first bottle, ease of twisting the lancet, warming the finger correctly, lancing the finger correctly and without fear, forming blood droplets of sufficient volume, getting the blood droplet into bottle 1, closing the cap of the first bottle correctly and pouring the contents of bottle 1,2 and 3 into the test device membrane correctly. In each case, the performance was between 83.14% and 99.71%. One participant needed assistance in all the steps despite primary level education and obvious ability to comprehend the instruction sheet.

### Readability results

To determine the capacity of lay users to properly interpret a range of test results, from invalid, weakly positive, positive and negative, sets of four non-functional devices prepared to display each type of this range of results were made available by the manufacturer. Ultimately, 91 subjects interpreted results of each type for these sets of devices used for the study. All the 91(100.00%) correctly identified positive results, negative results and invalid results without hesitation. However, when weakly positive dummy test results were provided, only 31 (34.07%) of the 91 were able to determine that these were weakly positive. The remainder, 60 (65.93%), were not sure what the results were.

### Laboratory performance results

At the close of the field study, 554 INSTI HIV self- tests were performed in the field. A total of 484 participants consented to venous blood draws; the samples were drawn successfully and made available for performance testing in the laboratory. From the 484, four (4) blood samples were found to have hemolyzed and were unsuitable for ELISA; data sheets from four (4) were found to be grossly incomplete and were not used for analysis. A total of 476 samples therefore had complete data.

From bioelisa HIV-1+2 Ag/Ab testing, 270 (56.72%) of the samples received were HIV negative and 206 (43.28%) were HIV positive. However, INSTI HIV Self-test showed 268 (56.30%) HIV negative, 202 (42.44%) HIV positive and 6 (1.26%) invalid results. All the six (6) invalid tests were from samples drawn from known HIV positive participants. The comparison of outcomes between bioelisa and INSTI HIV 1/2 self-test is provided in Table 2.

**Table 2:** Comparison of outcomes between bioelisa and INSTI HIV1/2 self-test.

	ELISA HIV Status		
INSTI HIV Self-test Results	Negative	Positive	Total
Negative	264(98.51%)	4(1.49%)	268 (100%)
Positive	6(2.97%)	196 (97.03%)	202 (100%)
Invalid	0 (0.00%)	6 (100%)	6 (100%)
Total	270(56.72%)	206(43.28%)	476 (100%)

### Performance characteristics of INSTI HIV Self-test in the hands of lay users

For purposes of determining sensitivity and specificity, the true HIV status was considered to be definitive when both ELISA and INSTI HIV Self-test were concordant. When the ELISA and INSTI HIV Self-test results were discordant, the final status was resolved using Alere Determine™ HIV 1/2 and First Response HIV-1-2 in line with the national algorithm.

The HIV status was positive for 207 (43.49%) samples and negative for 269 (56.51%) samples.

The six samples that returned invalid outcomes on the INSTI HIV Self-test tested positive both on ELISA and Alere Determine™ HIV 1/2. The invalid results were incorporated into the final sensitivity and specificity computation.

Six (6) samples tested positive on INSTI HIV Self-test but negative on ELISA. These samples were drawn from participants whose HIV status was unknown; three of the six tested negative on Alere Determine™ HIV 1/2.

Four samples tested negative on INSTI HIV Self-test and positive on ELISA. These samples were drawn from participants whose HIV status was unknown; two of these four samples were positive on Alere Determine™ HIV 1/2.

An exact McNemar’s test determined that there was no systematic difference between the two tests (p = 0.26).

Excluding invalid results, the sensitivity of the INSTI HIV Self-test was 98.99% (95% CI 96.05–99.75%), and the specificity 98.15% (95% CI 95.63–99.23%).Using this approach, the likelihood ratio positive was calculated as 0.9899/1-0.9815, giving a value of 53.51%. Inclusive of invalid results, the sensitivity was 96.10% (95% CI 92.37–98.04%), and the specificity 98.15%(95% CI 95.63–99.23%).The likelihood ratio positive was calculated as 0.9610/1-0.9815 giving a value of 51.95%. In both cases the concordance was 97.27%. These performance characteristics are summarized in Table 3.

**Table 3:** Performance characteristics of the INSTI HIV self-test in the hands of lay users.

	HCW Results		
Self-test Result	Negative	Positive	Total
**Negative**	266(98.15%)	2(0.98%)	268 (56.30%)
**Positive**	5(1.85%)	197 (96.10%)	202 (42.44%)
**Invalid**	0 (0.00%)	6 (2.93%)	6 (1.26)
**Total**	269 (100%)	207 (100%)	476 (100%)

### Determination of predictive values of the INSTI HIV Self-test in field settings in Kenya

The HIV prevalence in Kenya in 2015 was 5.9% [31]. We calculated the positive and negative predictive values (PPV and NPV respectively) of the INSTI HIV self-test using this prevalence to give a true reflection of how it would perform in field settings in lay hands. We found the PPV in Kenyan settings to be 77.04% and the NPV to be 99.94%.

## Discussion and conclusions

To the best of our knowledge, this is the first combined evaluation of the usability, readability, sensitivity, specificity, negative predictive value and positive predictive value of the INSTI HIV Self-test (bioLytical® Laboratories Inc., Richmond, B.C., Canada) using capillary blood and whole blood in field and laboratory evaluations done in Kenya.

At the close of the field study, 554 INSTI HIV self- tests were performed in the field. Despite that, only 484 venous blood samples were drawn successfully and made available for performance testing in the laboratory; 70 subjects did not provide consent for a venous blood draw. Ordinarily, these would not benefit from HIV testing in a centralized testing laboratory, but they could benefit from Voluntary Counselling and testing as well as from self-testing. Drawing of venous blood requires some skill, while venous blood samples are prone to hemolysis and can be damaged by improper storage conditions in transit. They are difficult to transport in hard to reach areas. From the 484 participants, four (4) blood samples were found to have hemolyzed and were unsuitable for ELISA. Self-testing would definitely benefit those who live in hard to reach areas and thereby improve Kenya’s chances of achieving the first 90 in UNAIDS 90-90-90 strategy in time.

For the usability study, more males than females were enrolled (206 and 142 respectively). This is a positive outcome from this study, the usual experience in sub Saharan Africa has been for more women to test for HIV than for men [32]. Our unusual finding may be related to the use of the county administration to mobilize participants. Also, scheduling of meetings was done on the days of village barazas (consultative meetings). Traditionally, these meetings are largely attended by men and not women, hence the large turnout for men.

Out of 350 participants for the usability study whose data was complete, 339 (96.86%) reported that it was easy to comprehend the instructions, while 330 (94.29%) found the test device easy to use. This was somewhat surprising, considering that 200 (57.14%) had only primary or no education. Actually, 46 (95.83%) of the 48 who had no education at all found the instructions for use easy to understand, and 42 (87.50%) found the test device easy to use. Literacy level of an individual is often measured by several years below what would be predicted by the number of years of schooling completed. Findings from health literacy studies demonstrate that literacy levels of patients in the health care setting is inadequate and low health literacy may occur even among well-educated patients. Additionally, requesting help to interpret and understand medical information is associated with fewer years of education and poorer numerical literacy skills [33–35]. On average, 38.5 per cent of the Kenyan adult population is illiterate. There are very wide regional disparities; for example, Nairobi had the highest level of literacy, 87.1 per cent, compared to North Eastern Province, the lowest, at 8.0 per cent [36]. The fact that a majority of illiterate people can use this device is very encouraging. One reason may well be that the instructions were well designed and clearly illustrated.

In fact, the lowest usability parameter in this study was in lancing the finger, where only 291(82.20%) participants were able to draw blood without fear. The culture in many parts of Kenya places significant importance on blood, and therefore this is not surprising.

On overall perception, 342 (96.61%) participants would use the test kit again, and 344 (97.18%) would recommended its use to a sexual partner. Similar studies have reported near universal acceptability (80–96%) of HIVST among the general population, with similar rates for men and women [37–39]. This is very encouraging and shows that the HIV testing landscape in Kenya has matured and is ready for self-testing.

Most family medical practitioners in a past study (Poirier et al 2015) stated that the test was difficult to implement in practice due to too few opportunities or requests for use, complex handling, difficulties in proposing the test, fear of having to announce seropositivity, and significantly longer consultation. It should be noted that in the past study, the family practitioners did not follow manufacturer’s instructions, although they received some training. To the best of our knowledge, no studies have been conducted locally to show usability in client’s hands. In this study, 349 (98.59%) participants were able to use the information sheet easily, and 303 (85.59%) were able to complete all steps of the test confidently. This high usability has been reported locally before [37] and compares favorably with Prazuck T. et al 2016 and Martinez Perez et al. 2017. It also implies that when the test is introduced in the market, a majority of people will be able to self-test and benefit from knowledge of their status with no further need to visit health care facilities.

**Fig 4:**
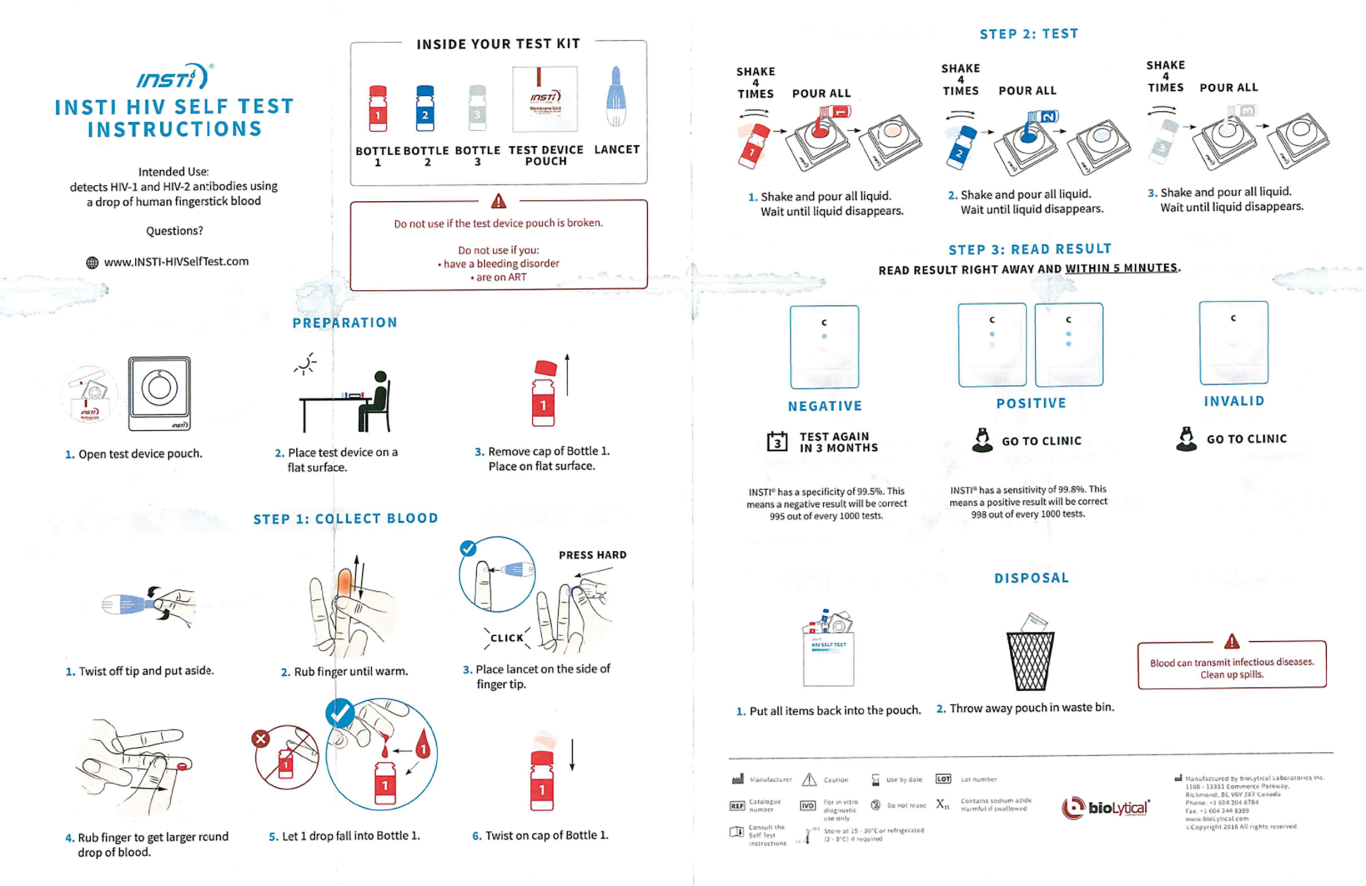
Instructions for use in English.

**Fig 5:**
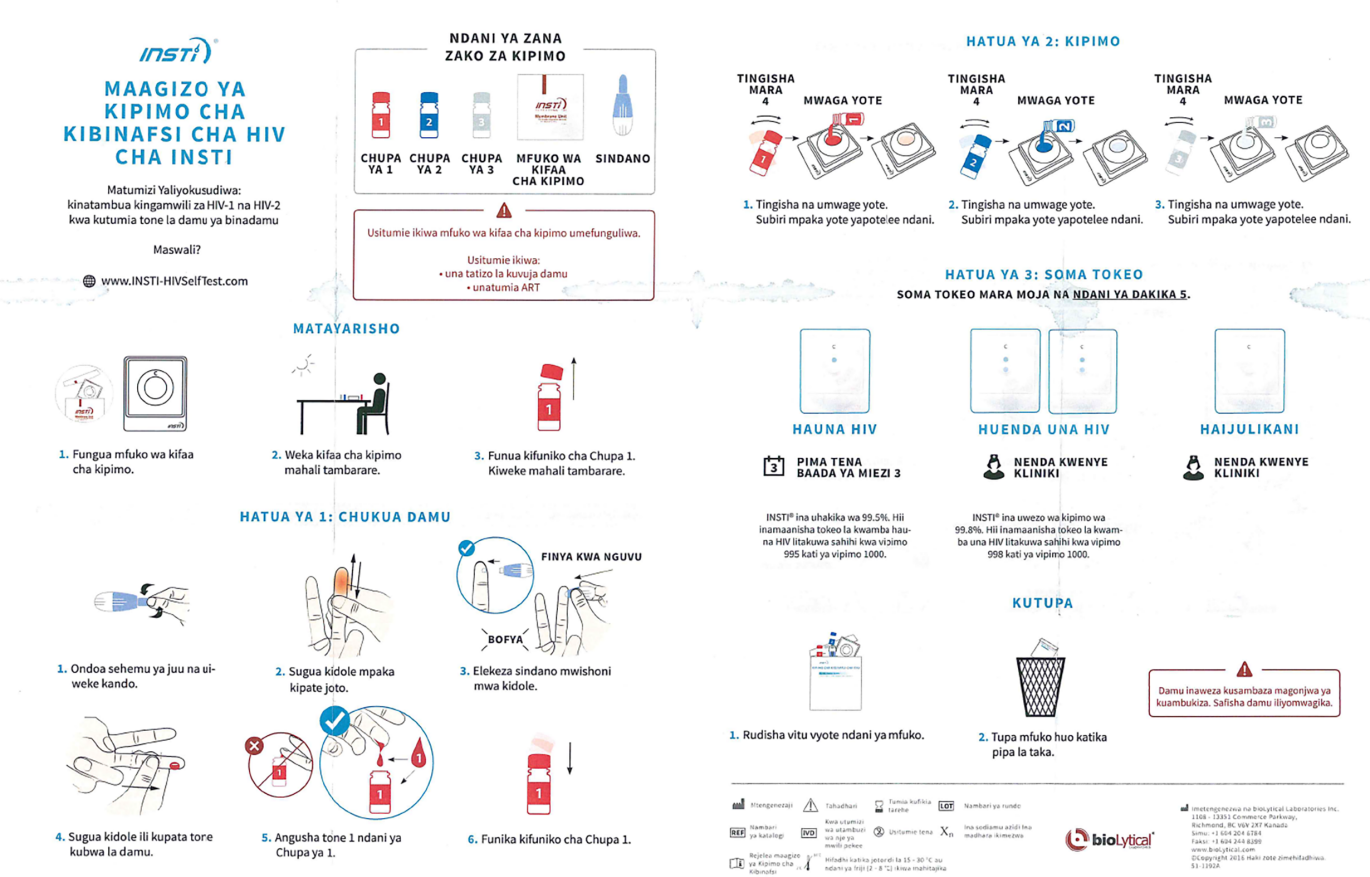
Instructions for use in Swahili.

On readability, whereas all the 91 participants correctly identified positive results, negative results and invalid results without hesitation, only 31 (34.07%) were able to identify weak positive results. When we reviewed the IFU that were provided with the INSTI HIV Self-test by the manufacturer, it was evident that the English version (Fig 4) showed images of strong and weak positive results correctly. The two illustrations provided in the Swahili version (Fig 5) of the instructions for the interpretation of positive results were both identical strong positive images. There was no weak positive image. These should also have been the illustrations in the Swahili page. This error from the manufacturer’s IFUs may have accounted for the uncertainty in interpretation of weak positive results.

Still, this is a significant limitation. In our experience, weak positive results are not common locally. It is necessary to provide additional support to the testing environment to ensure that weak positive results are identified correctly as Kenya moves towards self-testing. For instance, the manufacturer should add a phrase to each of the images to indicate that even a faint test spot is to be interpreted as positive.

The lack of bias between the reference bioelisa HIV-1+2 Ag/Ab test and I INSTI HIV Self-test is encouraging and suggests interchangeability. A HIV rapid diagnostic test (RDT) needs to have a sensitivity ≥70% and a specificity≥90% for it to be acceptable for use in routine self-testing settings.[40]Excluding invalid results, the sensitivity of the INSTI HIV Self-test was 98.99% (95% CI 96.05–99.75%), the specificity 98.15% (95% CI 95.63–99.23%) and the concordance 97.27% in lay hands. With a likelihood ratio positive of 53.51 and the result notification rate expected to be 100%, (as this is a self-test) the efficacy of this test is 53.51. Even if invalid results were to be included in the analysis, the likelihood ratio positive would be 51.95, and the efficacy in this case would be 51.95. With HIV prevalence approximating 5.9% in Kenya [31], the positive predictive value of this test in field settings is expected to be 77.04%. Combined with the high negative predictive value of 99.94%, this implies that the self-test has a significant role as a self-test in Kenya and can help improve access to testing.

Traditionally, ELISA results have been used to determine the true HIV status of a patient. In this study, discordant results between ELISA and INSTI HIV Self-test results were observed. In agreement with previous studies, additional tests such as a third rapid test as a tie-breaker, an enzyme-linked immunosorbent assay (ELISA) test for detection of antibodies and/or antigen, and HIV-RNA viral load testing should be used to determine the HIV infection status of an individual when the results of the screening and confirmatory tests are discrepant or give indeterminate results [41–43]. In this study, the final HIV status was resolved using Alere Determine™ HIV 1/2 and First Response HIV-1-2 kit (Premier Medical Corporation Ltd., Kachigam, India).

We also observed that six samples gave invalid results on the INSTI HIV Self-test but tested positive on ELISA. In our experience, invalid results are either related to quality control during the manufacturing process or insufficient sample volumes since the control is designed to only work when the correct sample volume is added to solution 1. It is noteworthy that we did not use blood transfer pipettes during this study; this may have led to insufficient sample volumes leading to invalid results. The final impact of transfer pipettes on invalid results, simplification of the test procedure and final retail prices should be investigated further. With strict adherence to good manufacturing practice by the manufacturer, and sufficient sample volumes, these errors can be reduced.

In the Kenyan program, it is anticipated that HIV self-tests will not be linked to counselling, but information on the steps to take after each test result will be provided in the instructions for use. A HIV positive result is often a traumatizing event and the potential for self-harm cannot be ruled out after HIV positive outcomes. Additionally, linkage to care may end up being lower than in the current testing setup.

While doing the field test, 2 test devices were observed to show delayed immunofiltration of buffer 1 after being stored at 29°C for a significant period. In spite of that, it is possible that the slow buffer flow rate is sample related; it is conceivable that lysed cells from certain samples can clog the pores of the membrane and result in a slow buffer flow rate.

In a study such as this, there is a real possibility of misclassification of a participant’s HIV status by ELISA or Determine or even both. No single assay has yet been designed to detect all possible HIV antigens and antibodies, and for that reason even a composite gold standard such as what we use remains susceptible to misdiagnosis. WHO recommends using an algorithm which uses rapid diagnostic tests (RDT) in combination to diagnose HIV[44] and hence reduce the possibility of misdiagnosis. There are other possible reasons for misclassifying HIV and these include cross reactivity across pathogens, administrative errors, poor test storage and transportation, poor quality control, insufficient training of users and inadequate supportive supervision of health care workers [45]. Many of these can be reduced significantly through quality control and assurance interventions.

Finally, the study team determined that there was significant soiling of hands due to the nature of economic activities undertaken by study participants. Although alcohol swabs were not provided by the kit manufacturer, the team decided to provide these. In field settings, proper washing of hands before the test can eliminate the need for alcohol swabs.

### Study limitations

The investigators used INSTI, Bioelisa and Alere Determine™ results in the final decision as to whether a sample was positive or not. This may have introduced incorporation bias into the study and could lead to an overestimation of diagnostic sensitivity and specificity. Given the absence of a generally widely accepted reference standard in the local testing landscape, this decision appeared reasonable[46] and is not uncommon for similar studies[38, 47]. In studies of laboratory tests, it is quite difficult to perform evaluations that are totally free of bias, a problem well described in literature [48–51]. Furthermore, western blotting has not been in use in Kenya as part of the reference standard. On further analysis, the inclusion of Alere Determine™ as the confirmatory test only improved the concordance marginally from to 96.64% to 97.27%.

The study participants used one to two drops of blood applied directly to the test cartridge. This approach differs from the practice common in the west where a capillary tube is used to place exactly 50ul of blood in the cartridge. From past experience, it is much more difficult to implement the use of capillary tubes in the Kenyan context even at voluntary testing and counseling centers, and the manufacturer appears to have taken that into consideration while developing the instructions for use. The absence of capillary tubes appears to have made the test less complicated to use and seems not to have affected the final outcomes. This is an advantage as capillary tubes may add to the cost and may increase the amount of hazardous waste generated.

Finally, the study team attempted to determine the predictive values of INSTI HIV self-test in field settings using a national prevalence of 5.9%. The exact prevalence of HIV for a particular population is the best way to determine PPV and NPV if known. In our settings, this is hardly feasible.

### Recommendations

The usability, sensitivity and specificity of the INSTI HIV Self-test has met and exceeded the minimum usability standards set for environments with a generalized HIV epidemic. Intended users in diverse populations could benefit from its high sensitivity and specificity as well as excellent usability. It can help improve access to testing in rural areas and in areas where stigma is still high.

## Annex 1: Questionnaire for the observer

1. Did the study participant read/use the information sheet?
2. Was it difficult for the study participant to remove the test device from the pouch?
3. Was the study participant able to remove the cap of Bottle 1?
4. Did the study participant twist the tip of the lancet off?
5. Did the study participant rub his/her finger correctly (up and down/vertical motion)?
6. Was the study participant able to lance his/her finger correctly?
7. Was the study participant able to form a blood droplet?
8. Was the study participant able to get the blood droplet to fall into Bottle 1 as a free flowing drop of blood?
9. Did the study participant wipe the blood droplet from the finger on the rim of Bottle 1 to get the blood into the Bottle?
10. Was the study participant unable to get any amount blood into Bottle 1?
11. Was the study participant able to twist the cap onto Bottle 1?
12. Did the study participant shake Bottle 1, 4 times?
13. Did the study participant pour the liquid from Bottle 1 into the membrane unit and wait until liquid disappeared?
14. Did the study participant shake Bottle 2, 4 times?
15. Did the study participant pour the liquid from Bottle 2 into the membrane unit and wait until liquid disappeared?
16. Did the study participant shake Bottle 3, 4 times?
17. Did the study participant pour the liquid from Bottle 3 into the membrane unit and wait until liquid disappeared?
18. Did the participant quit the process at any point?
